# Aggressive fibromatosis of the oral cavity in a 5 year old boy: a rare case report

**DOI:** 10.11604/pamj.2017.27.47.11739

**Published:** 2017-05-18

**Authors:** Keerthi Krishnankutty Nair, Kanad Chaudhuri, Ashok Lingappa, Ranjani Shetty, Pramod Gujjar Vittobarao

**Affiliations:** 1 Department of Oral Medicine and Radiology SJM Dental College and Hospital, Chitradurga, Karnataka, India; 2 Dental Surgeon, Lifeline Polyclinic Kalyani, Nadia, West Bengal, India; 3 Department of Oral Medicine and Radiology Bapuji Dental College and Hospital MCC B Block, Davangere, Karnataka, India

**Keywords:** Aggressive fibromatosis, desmoid tumor, infantile fibromatosis, soft tissue tumor, extra-abdominal fibromatosis

## Abstract

Fibrous tissue proliferations express a wide spectrum of histologic and morphologic variation in both infants and adults. This ranges from hypertrophic scar formation at one end to malignant fibrosarcoma at the other end of the spectrum. Aggressive fibromatosis is an intermediate tumor which is in proximity to fibrosarcomas. These are locally invasive and often recur after excision, but do not metastasize. Histologically, they are characterized by proliferating fibroblasts with little mitotic activity. Aggressive fibromatosis in the head and neck region is not common, and very sporadically occurs in the oral cavity or jaw bones. Here we report a rare case of aggressive fibromatosis occurring in a 5 year old boy.

## Introduction

Fibromatoses encompass many different entities of well-differentiated fibroblastic proliferations showing variable collagen production and formation of a firm nodular mass [1]. These tumors are intermediate in clinical, radiological, microscopic presentation and biologic potential to that of benign and malignant tumors. It is considered locally aggressive because of its infiltrative growth pattern, but does not metastasize. Aggressive fibromatosis in the head and neck region is not common, and very rarely occurs in the oral cavity or jaw bones [2]. Infantile desmoid fibromatosis is considered as a paediatric equivalent of adult musculo-aponeurotic desmoid fibromatosis and predominantly occurs in male patients below 8 years of age [3].

## Patient and observation

**Figure 1:**
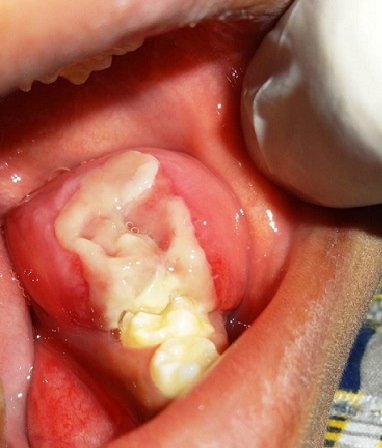
A solitary soft tissue mass on the left posterior mandible over the alveolar ridge distal to 75 with superior surface showing ulceration due to occlusal trauma from 65

A 5 year old boy reported to our department with a complaint of painless swelling in the left side of the mouth since 2 months. The swelling was peanut size to begin with, which gradually increased to the present size and was not associated with fever or weight loss. There was no associated history of trauma. His past medical and dental history were non-contributory. On examination right and left submandibular lymph nodes were palpable, measuring 1x1cm, firm, mobile and tender on palpation. A diffuse swelling on the left posterior aspect of the jaw caused mild asymmetry of the face. Intra oral examination revealed a complement of primary dentition. A solitary, sessile, soft tissue mass was present on the left posterior mandible over the alveolar ridge distal to 75, measuring approximately 2.5cm x 3cm, roughly oval in shape. The antero-posterior extent was from the middle of the left primary 1^st^ molar to the pterygomandiular raphe region, and bucco-lingually from the depth of the buccal vestibule to the muco-gingival junction on the lingual aspect. The surface of the swelling was smooth except for an ulcer on the superior surface, caused due to occlusal trauma from 65. The mass was firm in consistency, non-tender and mobile on a broad base. There was mild bleeding on manipulation (Figure 1). A provisional diagnosis of benign soft tissue tumor on left mandibular alveolar ridge was made and differential diagnoses of peripheral ossifying fibroma, peripheral giant cell granuloma & peripheral odontogenic fibroma were considered for the soft tissue mass.

**Figure 2:**
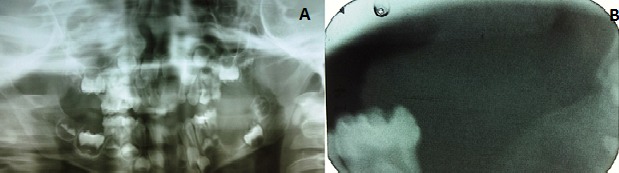
(A) OPG revealing an area of moderately well-defined radiolucency on the left posterior mandibular alveolar ridge distal to 75 with a discontinuous sclerotic margin; a speck of calcified mass was evident on the pericoronal region of the developing 36; (B) IOPA radiograph in the area revealed a soft tissue shadow and evidence of calcification with radiodensity similar to dentin

**Figure 3:**
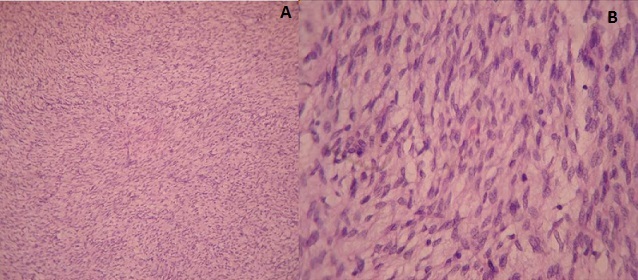
(A) H&E 40X & (B) H&E 100X: histopathological section revealing spindle cells in fascicles and parallel to blood vessels at places; typical 5-10 mitosis/10 hpf were seen with no abnormal mitotic figures

**Figure 4:**
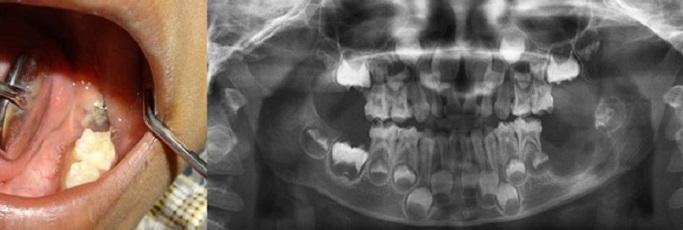
14^th^ day post-operative clinical picture and radiograph (OPG)

**Figure 5:**
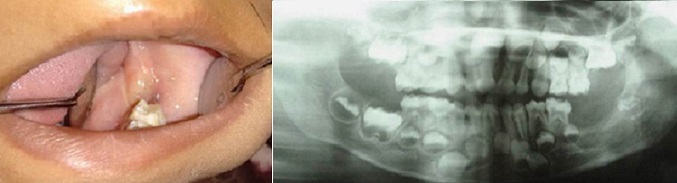
3 months post-operative clinical picture showing satisfactory healing & radiograph (OPG)-revealing partial filling of the surgical bone defect

Panoramic radiograph revealed a complement of primary dentition and developing permanent dentition. An area of moderately well-defined radiolucency was evident on the left posterior mandibular alveolar ridge distal to 75 with a discontinuous sclerotic margin. The developing tooth bud of 36 was inferiorly displaced (Figure 2A). A speck of calcified mass was evident on the pericoronal region of the developing 36. Intra oral periapical radiograph in the area revealed a soft tissue shadow and evidence of calcification with radiodensity similar to dentin (Figure 2B). Complete hemogram was done and all parameters were within the normal limits. Excision of the mass along with developing tooth bud of 36, followed by curettage was done. The lesion appeared to have some attachment to the crown of the developing tooth bud. The excised mass was 1*2*3 cm in dimension, grayish white with ulceration. Another soft tissue mass of 1*2*1 cm was attached to the tooth. The histopathological examination revealed ulcerated mucosa overlying an unencapsulated lesion composed of spindle cells in fascicles and parallel to blood vessels at places. Typical 5-10 mitosis/10 hpf were seen. The section from the soft tissue attached to the tooth showed rests of odontogenic epithelium surrounded by mesenchymal tissue and dentin (Figure 3A, Figure 3B). Based on this a final diagnosis of the aggressive fibromatosis of left side mandibular alveolar ridge was made. On the 14th postoperative day, sutures were removed & a slow healing was observed (Figure 4). Knowing the aggressive nature of the tumor, patient was kept under follow- up every 3 months. 3 months follow up radiograph revealed partial filling of the surgical bone defect (Figure 5).

## Discussion

Aggressive fibromatoses are distinctive lesions which are defined as a group of non-metastazing fibrous tumors which tend to invade locally and recur after surgical excision [4]. It is thought to develop from the tissue of the musculo-aponeurotic system [2]. Many other terms exist for this entity, including extra-abdominal fibromatosis, desmoid tumor, well differentiated non metastasizing fibrosarcoma and grade I fibrosarcoma [5]. The classification of fibromatosis has not yet been standardized. Two principal forms are recognized-juvenile fibromatosis and adult fibromatosis [4]. Based on the site of involvement it has been classified as abdominal fibromatosis and extra-abdominal fibromatosis. Extra-abdominal tumors are predominantly sporadic [6]. Abdominal fibromatosis is characteristic because of its location and its tendency to occur in women of childbearing age especially during or following pregnancy. Abdominal fibromatosis in children is an extremely rare condition [1]. Enzinger and Weiss classified them broadly into superficial & deep fibromatosis [7]. Desmoplastic fibroma is a very rare primary bone tumor morphologically resembling desmoid-type fibromatosis and is considered as its osseous counterpart [8]. The exact etiology of aggressive fibromatosis is unknown. The physical factors such as trauma; including surgical trauma and radiation are said to have some association with the development of the tumor [2]. Endocrine factors have been implicated since the progression of the tumor appears to be hormonally based. Higher incidences during & after pregnancy and following exposure to oral contraceptives as well as spontaneous regression during menopause support this association [9]. Though a viral theory has also been suggested, no virus has been isolated [2]. A genetic predisposition has been suspected considering the association with familial adenomatous polyposis (FAP) and Gardner´s syndrome [10]. FAP is an autosomal dominant condition resulting from germline mutation of the adenomatous polyposis coli (APC) gene. Desmoid tumors are 10-20% prevalent in FAP with a frequency approximately 850 times that of the general population [10, 11]. Aggressive fibromatosis may involve a deregulation of connective tissue growth and it has been suggested that the process is neoplastic rather than reactive [9]. The deep fibromatosis is suggested to have somatic β-catenin or APC gene mutations causing increased intranuclear accumulation of β-catenin that may result in proliferation of cells [12]. At the same time the tumor suppressor gene Rb1 has been shown to have decreased expression and may also play a role in tumor progression [13]. Nuclear β-catenin expression has been suggested as a tumor-specific marker for aggressive fibromatosis. However Sharma A (2008) conducted a retrospective study in a total of 10 paediatric patients with aggressive fibromatosis of the head & neck and found that 4 cases (40%) were positive and 6 cases (60%) were negative for nuclear β-catenin expression. This study concluded that nuclear β-catenin expression should not be considered a specific tumor marker for paediatric aggressive fibromatosis of the head and neck [9].

Annual occurrence of aggressive fibromatosis is 0.2-0.4 per 100,000, with origin in the head and neck accounting for 10-25%. Since 1954, 97 cases of paediatric head and neck fibromatosis have been reported. The age range was from birth to 16 years, with the average being 4 years and 5 months. The majority of tumors were found in the mandible (38%) [14]. In the oral cavity, it most often presents as a painless mass or swelling in the region involved. Less often it causes pain or discomfort, trismus, ulceration, intraoral bleeding, otalgia, dysphagia, dyspnea, and loose teeth. These signs and symptoms vary depending on the location. The average duration from onset to diagnosis is 3.4 months & average tumor size was 3.8 cm in largest dimension [7]. Aggressive fibromatosis has a locally infiltrative nature and manifests as a secondary erosion or invasion of the bone [7]. In a conventional radiograph it appears as a poorly defined radiolucency. Along with local erosion it may cause periosteal reaction, deformation, or bowing of bone. Conventional radiography only gives a rough estimate of tumor size and location [3]. Fine needle aspiration biopsy is typically insufficient to arrive at a specific diagnosis [15]. Sonographic appearances are nonspecific. Solid lesions frequently appear homogeneous and hypoechoic but rarely show heterogeneous and variable echogenicity. A sonography guided biopsy can be performed in case of superficial lesions [3]. The computed tomograhic appearance of infantile desmoid fibromatosis is variable and nonspecific. Plain CT and contrast-enhanced CT provide reliable information about anatomic relationships and tumor extent; however the margins of some lesions may be poorly defined. Attenuation of most tumors are similar to that of muscle or slightly increased. CT using bone window settings allows more accurate assessment of bone involvement and destruction than conventional radiography or MR imaging. The lesion becomes obvious after the injection of iodinated contrast material, but with varying degree of enhancement. CT guided biopsy can be performed for deep lesions [3]. The superior soft-tissue contrast of MR imaging, its multiplanar capabilities, and its lack of beam-hardening artefacts allow accurate tumor delineation and a greater appreciation of infiltration of adjacent structures than does CT [3]. The variable MR imaging signal characteristics of infantile desmoid fibromatosis reflect differences in composition. Most desmoid tumors are heterogeneous soft-tissue lesions of intermediate signal intensity between those of muscle and fat [16]. Zones of low signal intensity are often seen on both T1- and T2-weighted images. These areas reflect hypocellularity and abundant collagen whereas tumors with high cellularity and abundant collagen show increased signal intensity on T2-weighted images. The differences in T2-weighted signal intensity appears to be determined by the degree of cellularity rather than the amount of collagen present in the lesion. After the IV injection of gadolinium, tumors may show homogeneous, non-homogeneous or no significant enhancement [3]. Biopsy is essential, because only histological examination can distinguish between the various forms of the tumor and is important for differential diagnoses [5]. Tumors may be composed of immature mesenchymal cells or more mature fibroblasts arranged in bundles or fascicles with varying amounts of collagen with rare atypia and mitoses [2]. Among reported cases of paediatric aggressive fibromatosis of head and neck, 74% underwent large surgical resection as the primary treatment modality. 16% of these tumors reported recurrence [14]. Wang et al (2014) proposed an age based treatment of aggressive fibromatosis of head & neck. He suggested that tumors which can be resected with negative margins without functional & cosmetic impairment should undergo surgical resection as a primary treatment modality. If the surgical resection causes impairment, then a conservative resection has to be done with chemotherapy in patients less than 18 years of age and radiotherapy in patients more than 18 years. All the cases should have scheduled follow up regardless of the treatment modality. There is however a positive correlation between clear histologic margins and long-term disease-free survival [17].

## Conclusion

Fibromatosis in maxillofacial region is a very rare occurrence, and because of its rarity, criteria for clinical diagnosis and treatment protocols have not been established. This could be a possible reason for high recurrence rate.

## Competing interests

The authors declare no competing interests.
